# The impact of parasitic diseases on dromedary camel (*Camelus dromedarius*) welfare: a comprehensive review

**DOI:** 10.3389/fvets.2025.1732337

**Published:** 2026-01-23

**Authors:** Saqib Nawaz, Mohamed Tharwat

**Affiliations:** 1 Anhui Province Key Laboratory of Veterinary Pathobiology and Disease Control, College of Veterinary Medicine, Anhui Agricultural University, Hefei, China; 2 Department of Clinical Sciences, College of Veterinary Medicine, Qassim University, Buraidah, Saudi Arabia

**Keywords:** animal welfare, dromedary camel, one health, parasitic diseases, pastoralism

## Abstract

The dromedary camel (*Camelus dromedarius*) is a multifunctional animal indispensable for the livelihoods and food security of pastoralist communities residing in arid and semi-arid regions. Despite its socio-economic and cultural significance, the welfare of camels has garnered limited scientific scrutiny, particularly regarding the effects of parasitic diseases. These diseases pose a significant barrier to camel health, resulting in considerable production losses and severe welfare challenges. This review consolidates evidence on the impact of parasitic infections, which include hemoparasites (*Trypanosoma evansi*, *Babesia*, and *Theileria*), ectoparasites (ticks and mange mites), and endoparasites (gastrointestinal nematodes and *coccidia*) on the welfare of dromedary camels. We investigate the physiological and emotional repercussions of parasitism through the lenses of the Five Freedoms and the Five Domains model. This review demonstrates that parasitic diseases severely impact camel welfare, yet it highlights significant deficiencies in the species-specific assessment and surveillance systems needed to address these problems. Furthermore, it underscores the relationship between camel welfare, human health through zoonotic parasites, and the socio-economic stability of pastoral communities. The review concludes that an integrated, multidisciplinary approach combining veterinary parasitology, animal welfare science, and socioeconomics is urgently required. We advocate for the implementation of a cohesive One Health/One Welfare framework to establish validated welfare indicators, enhance diagnostic and control strategies, promote community engagement, and inform effective policies. This strategy is crucial for alleviating suffering, improving productivity, and sustaining livelihoods that depend on camels in the face of climate change.

## 1 Introduction

The dromedary camel (*Camelus dromedarius*) is a species specially adapted to thrive in arid and semi-arid environments, where it plays an essential role in providing resilience against water shortages, extreme temperatures, and limited grazing opportunities (1). Its physiological adaptations, such as efficient water metabolism, exceptional heat tolerance, and specialized movement across sandy terrains, enable it to survive in conditions where other livestock cannot. These traits have supported the livelihoods of pastoralist communities across North Africa, the Middle East, and Asia for thousands of years (2). Today, approximately 35 million dromedary camels are distributed across 47 countries, underscoring their enduring social and economic importance (3). The camel’s value extends beyond transport to being a crucial source of milk, meat, and income, thereby underpinning the food security and economic resilience of pastoralist communities (4). Its deep cultural significance further cements its role in the social fabric of these regions (5).

However, despite their socio-economic value, dromedary camel welfare remains a critically under-researched area, with parasitic diseases representing a particularly neglected aspect (6). Key knowledge gaps persist, including: a lack of validated, species-specific welfare indicators tailored to camels; an incomplete understanding of the affective states (e.g., pain, itching, distress) induced by different parasites; and insufficient integration of parasitology and welfare science within a cohesive framework that also considers human and environmental health (6, 7).

A major obstacle to dromedary camel welfare is their high susceptibility to a wide range of parasitic infections. Assessing welfare in this species is challenging due to extensive management systems and limited veterinary infrastructure (8), creating a pressing need for validated, camel-specific assessment protocols (9). Parasitic diseases exacerbate this challenge by causing not only production losses but also significant, often overlooked, welfare compromises (8). This review, therefore, aims to synthesize current evidence on the multifaceted impact of parasitic diseases on dromedary camel welfare (10). We will examine how parasitism influences key welfare domains (nutrition, physical health, behavior, and mental state) while also considering the broader implications for pastoralist livelihoods and public health through zoonotic transmission (3). By integrating insights from disease ecology, welfare assessment, and socio-economics, this review is structured around a cohesive One Health/One Welfare approach, highlighting the inextricable links between animal, human, and environmental well-being (11).

## 2 Parasitic diseases and animal welfare

**Figure 1:**
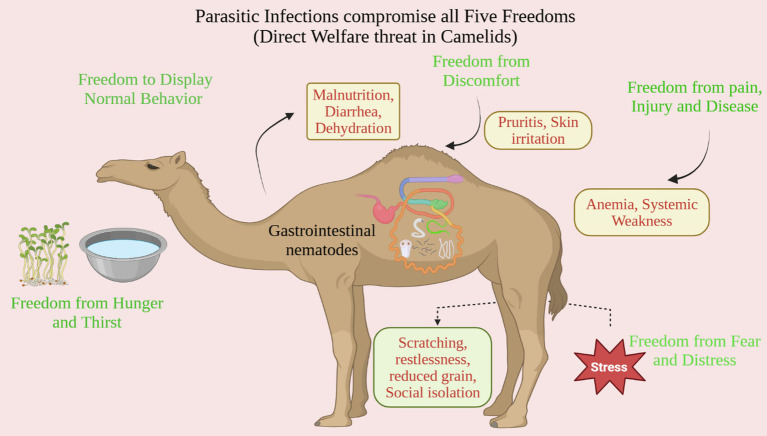
Schematic illustration showing how parasitic infections compromise the five freedoms of animal welfare in camelids. The diagram emphasizes the direct pathways through which common parasites, especially gastrointestinal nematodes, impact welfare: causing discomfort (pruritus, skin irritation), pain and disease (anemia, systemic weakness), hunger and thirst (malnutrition, diarrhea, dehydration), and ultimately resulting in abnormal behaviors (scratching, restlessness, social isolation) as well as fear and distress.

Animal welfare encompasses the overall well-being of an animal, including its physical health, mental state, and ability to perform natural behaviors. The Five Freedoms framework highlights essential rights, such as freedom from hunger and thirst, discomfort, pain, injury, and disease, as well as fear and distress, and the freedom to exhibit normal behavior (4). Building on this, the more recent Five Domains model offers a detailed method to assess welfare through nutrition, environment, health, and behavior, with the fifth domain emphasizing the animal’s mental state and subjective experiences (12). Parasitic diseases pose a serious threat to each of the Five Freedoms in camels. First, the freedom from hunger and thirst is undermined as gastrointestinal nematodes compete for nutrients and can cause diarrhea, resulting in malnutrition and dehydration (13, 14). Moreover, parasites like mange mites cause intense skin irritation, infringing on the freedom from discomfort by causing persistent itching. Even more concerning, infections such as *Trypanosoma evansi* can cause anemia and systemic weakness, challenging the freedom from pain, injury, and disease (15, 16). The freedom to express normal behavior is also affected, as tick or mange mite infestations lead to excessive scratching and restlessness, reducing grazing time and possibly leading to social isolation (16, 17). Lastly, the chronic pain and suffering from untreated parasitic infections, along with the stress of delayed treatment or culling, compromise the freedom from fear and distress (18). Therefore, controlling parasites effectively is not just a production concern but an essential part of protecting the overall welfare of camelids (19) (Figure 1).

Implementing these models for dromedary camels requires context-specific modifications, which include extensive nomadic pastoralism, limited access to veterinary care, and ongoing exposure to parasites and extreme climates (20). Parasitic infections cause a range of welfare concerns through both direct physiological harm and indirect behavioral effects. For example, gastrointestinal nematodes interfere with nutrient absorption, leading to weight loss, diarrhea, and chronic dehydration (21). Ectoparasites, such as ticks and mange mites, cause severe itching, skin scratching, and lesions, which lead to restlessness and discomfort. These infestations trigger chronic stress responses that may weaken immune function and reduce overall resilience (5). Hemoparasitic infections, like those caused by *Trypanosoma evansi*, result in anemia, significant lethargy, and generalized weakness, clearly indicating serious welfare problems (22). The clinical signs of these parasitic infections often include decreased mobility, social withdrawal, and excessive scratching, which disrupt natural herd dynamics and social structures (23). Chronic diseases, especially when combined with poor management practices, cause prolonged stress, further lowering both welfare and productivity (9).

A comprehensive welfare assessment in dromedary camels requires developing and validating species-specific Animal-Based Measures (ABMs). Recent protocols highlight several important indicators (24), including Body Condition Score (BCS), coat quality, and the presence of skin lesions or ocular discharge, which reflect nutritional status and parasite burden (9, 25, 26). Also noted are signs such as abnormal scratching, reduced rumination, social withdrawal, and decreased grazing efficiency (10, 25). Anemia, evaluated through packed cell volume, and the quantitative measurement of parasite load in blood or feces, are also crucial (27, 28). Pastoralists often observe subtle signs of distress like decreased appetite, reduced milk production, or reluctance to walk, providing essential context (29).

Nevertheless, the consistent implementation of these ABMs faces considerable obstacles within extensive camelid production systems. The nomadic management across expansive territories complicates regular clinical assessments. Moreover, the limited availability of veterinary services in remote regions leads to substantial underreporting and delays in the treatment of parasitic infections (9, 30). Cultural attitudes that perceive parasites as an unavoidable burden often postpone intervention until clinical manifestations become severe (23). A significant drawback is the absence of universally standardized and validated welfare protocols specific to camels, in contrast to those developed for cattle and small ruminants (8, 31). This situation often renders the pain caused by chronic, debilitating conditions (9) like hemoparasitism ‘invisible,’ leading to a pervasive underestimation of suffering (12, 32) (Table 1).

**Table 1:** Validated and potential animal-based measures (ABMs) for welfare assessment in dromedary camels.

Welfare domain	Animal-based measure (ABM)	Description	Association with parasitic disease	References
Nutrition	Body condition score (BCS)	5-point or 9-point scale assessing fat and muscle cover over ribs, spine, and pelvis.	Low BCS is directly linked to GI nematodes, trypanosomiasis (chronic form).	(103)
	Camel grimace scale (Potential)	Facial expression scoring for pain (e.g., orbital tightening, ear position, muzzle tension).	Validated in other livestock; high potential to assess pain from mange and lameness.	(20, 104)
Health	Packed cell volume	Percentage of red blood cells in blood; measured via microhematocrit.	Key indicator for hemoparasites (e.g., *T. evansi*, *Anaplasma*) and hematophagous parasites.	(105)
	Skin lesion score	Quantitative assessment of area and severity of lesions (e.g., from mange, ticks).	Directly measures the impact of ectoparasites.	(106)
	Ocular/nasal discharge	Presence/absence or severity score of abnormal discharge.	It can indicate general systemic illness or specific infections.	(107)
Behavior	Scratch index	Number of scratching bouts per unit time (e.g., per 15 min).	Direct indicator of pruritus from mange mites, lice, or ticks.	(108)
	Grazing/rumination time	Duration of grazing and rumination is measured by observation or sensors.	Reduced time indicates lethargy (trypanosomiasis) or discomfort.	(109)
	Posture and activity	Qualitative assessment (e.g., lethargic, alert) or time spent standing/lying.	Lethargy and recumbency are common in severe anemia or systemic illness.	
Mental state	Avoidance distance	The distance at which a camel moves away from an approaching human.	Increased distance can indicate fear or distress due to chronic pain/poor handling.	(110)
	Qualitative behavioral assessment (QBA) (Potential)	Holistic assessment of expressive behavior (e.g., “content,” “agitated,” “depressed”).	Can capture the affective state resulting from chronic parasitic burden.	

A comprehensive assessment, viewed through the perspectives of the Five Freedoms and the Five Domains model, indicates that parasitic diseases pose a significant welfare issue, adversely affecting nutrition, health, behavioral expression, and mental well-being. The complex management systems inherent in camel husbandry exacerbate these shortcomings by hindering prompt diagnosis and effective intervention. Consequently, integrating the fields of veterinary parasitology and animal welfare science is not only advantageous but crucial for a complete understanding of camel health. Future initiatives should prioritize the development of validated, practical welfare assessment tools that are tailored to the unique physiological and ethological needs of the dromedary camel, ultimately facilitating improved management, timely treatment, and enhanced well-being within a One Welfare framework (25, 33).

## 3 Overview of parasitic diseases in dromedary camels

Parasitic infestations pose a significant challenge to the health of dromedary camels, severely restricting their productivity, physiological resilience, and overall welfare. These pathogens are typically categorized into hemoparasites, ectoparasites, and endoparasites, each causing unique yet frequently overlapping clinical and subclinical impacts. The resulting multifactorial impairments encompass reduced milk yield, suboptimal body condition, reproductive failures, and diminished work capacity, alongside direct welfare issues such as chronic pain, itching, anemia, and behavioral distress.

### 3.1 Hemoparasitic infections

#### 3.1.1 Trypanosomiasis (Surra)

*Trypanosoma evansi*, the causative agent of Surra, is a highly significant hemoparasite that is mechanically transmitted by blood-feeding flies such as *Tabanus* and *Stomoxys* spp. (34). The clinical symptoms include progressive anemia, cachexia, intermittent fever, and dependent edema (35–37). The economic repercussions of Surra are substantial, primarily due to decreased productivity rather than elevated mortality rates. In untreated outbreaks, mortality can reach 10–20%, but the morbidity is often significantly higher, impacting as much as 30–50% of a herd, which leads to chronic debilitation (2, 38). Infected camels experience severe weight loss, which can decrease meat and milk production by 15–30%, and their draft capacity may be reduced by more than 50%, severely affecting the livelihoods of pastoralists (39). Chronic infections often result in abortion, infertility, and high mortality if left untreated. From an animal welfare standpoint, Surra causes significant weakness, greatly diminishes foraging ability and mobility, and leads to chronic distress, making it one of the most debilitating diseases affecting camels worldwide (35, 39).

#### 3.1.2 Babesiosis

Babesiosis is an emerging tick-borne disease in dromedary camels, caused by protozoan parasites belonging to the genus *Babesia* (for instance, *Babesia caballi* and *B. canis*). Serological and molecular investigations have uncovered a surprisingly high prevalence of the disease, with rates varying from 5% to over 20% in certain regions of Africa and the Middle East, suggesting that endemic transmission is frequently underestimated (40). The pathogenicity of Babesia in camels can be considerable, although it differs depending on the species and the host’s immune response. Clinical manifestations of babesiosis include high fever, hemolytic anemia, hemoglobinuria (characterized by reddish urine), icterus, and lethargy (41). In acute instances, mortality rates can be significant, ranging from 10 to 30%, especially among young, elderly, or stressed animals, while morbidity within an exposed herd can also be elevated (42). The economic implications are considerable, arising from direct mortality, treatment expenses, production losses due to anemia and weight reduction, and diminished work performance. Additionally, infected camels may become chronic carriers, acting as reservoirs for ticks and sustaining the infection cycle, which complicates control measures (2, 43).

#### 3.1.3 Theileriosis

Theileriosis is an economically significant tick-borne hemoparasitosis in camels, caused by various *Theileria* species, such as *Theileria camelensis* and *T. annulata*. While certain infections may remain subclinical, pathogenic strains can lead to a severe condition known as tropical theileriosis (34). Studies on prevalence indicate notable geographic differences, with infection rates ranging from 10 to 50% in endemic regions, as identified through molecular techniques (44). The clinical manifestations are similar to those observed in bovids and include symptoms such as high fever, anorexia, lymphadenopathy, progressive anemia, and edema. The disease can be lethal, with mortality rates reported between 5 and 20% in naive herds, while morbidity can significantly impact a large segment of the herd, resulting in widespread debilitation. The economic ramifications are complex, encompassing mortality, decreased milk and meat production, loss of draft power, and costs associated with acaricides and chemotherapy (45). The implications for animal welfare are severe, involving direct suffering from febrile illness and anemia, as well as the stress linked to clinical disease and its treatment.

#### 3.1.4 Anaplasmosis

Anaplasmosis is caused by *Anaplasma marginale* and related rickettsial species that infect erythrocytes, resulting in hemolytic anemia, icterus, and systemic debilitation (46). Transmission is facilitated by tick vectors, with seroprevalence rates showing considerable geographic variation, ranging from 10% to over 60% in certain herds, indicating widespread exposure (46, 47). While morbidity in naive herds can be significant, mortality rates are generally low, approximately 1–2%; however, the economic impact arises from chronic production losses. Infected camels frequently become persistent carriers, experiencing reduced weight gain, lower milk production, and increased vulnerability to other diseases, which collectively impose considerable financial burdens on camel owners (47, 48). The welfare consequences include chronically diminished stamina, suffering related to persistent anemia, and increased susceptibility to secondary infections.

### 3.2 Ectoparasites

#### 3.2.1 Ticks

Ixodid ticks (*Hyalomma*, *Rhipicephalus*, *Amblyomma* spp.) are widespread ectoparasites and carriers of significant pathogen groups (49–52). Severe infestations result in direct harm through skin damage, abscess development, and severe irritation, leading to restlessness and excessive scratching (53, 54). The economic repercussions are diverse. An individual adult tick can consume 0.5–2 mL of blood, and substantial infestations can result in considerable blood loss, leading to anemia and reduced productivity (55, 56). Annual losses attributed to ticks are estimated to include a 16% decrease in milk yield and a 11% decline in weight gain, which critically impacts pastoralist economies (57). Additionally, tick-borne diseases (such as theileriosis and anaplasmosis) exacerbate these losses (58). The dual function of ticks as both direct parasites and disease vectors significantly enhances their welfare and economic consequences (57).

#### 3.2.2 Mange mites

Sarcoptic mange (*Sarcoptes scabiei* var. *cameli*) is a highly debilitating and contagious skin disease. Its prevalence can reach up to 25% in affected herds, with morbidity soaring to 100% if not managed effectively (59). Although direct mortality rates are low, the economic ramifications are severe due to substantial production losses. Camels affected by this condition may suffer a reduction of up to 40% in hide value, a 30% drop in milk production, and notable weight loss due to severe itching and stress (59, 60). The intense itching can lead to alopecia, lichenification, and self-inflicted injuries that increase the risk of secondary bacterial infections. Affected camels experience considerable pain and discomfort, often resulting in social withdrawal from the herd due to changes in behavior. This condition is rightly considered one of the most pressing welfare concerns in the management of camelids (59).

### 3.3 Other ectoparasites

Infestations caused by lice (*Bovicola* spp.) and flies that induce myiasis (such as *Wohlfahrtia magnifica*) play a significant role in dermatological issues, pruritus, and stress. Lice infestations may result in restlessness, severe cases can lead to anemia, and they can also damage hair fibers, thereby diminishing the quality of wool (61). Myiasis, which refers to the invasion of living tissue by fly larvae, can result in painful lesions, secondary infections, and, if not treated, can be fatal (49). Although often neglected, these parasites exacerbate welfare issues and lead to production losses, especially in animals that are already weakened by co-infections or inadequate nutrition (62, 63).

### 3.4 Endoparasites

#### 3.4.1 Gastrointestinal nematodes

Strongylid nematodes, including *Haemonchus*, *Trichostrongylus*, and *Ostertagia*, along with whipworms (*Trichuris*), are notably widespread, with prevalence rates frequently surpassing 70–90% in pastoral herds, thus establishing them as arguably the most prevalent parasitic challenge (64). These parasites lead to gastroenteritis, diarrhea, weight loss, and anemia. The economic repercussions are considerable, primarily due to diminished feed conversion efficiency, stunted growth in juvenile camels, and reduced milk yield in lactating females. *Haemonchus longistipes*, a particularly virulent species, can induce significant blood loss, resulting in anemia and bottle jaw, which diminishes the market value of the affected animals (61, 65). The main welfare issues arise from chronic malabsorption and protein depletion, which lead to ongoing hunger, dehydration, and decreased vitality, ultimately impairing mobility and reproductive behaviors (2, 64).

#### 3.4.2 Coccidiosis

Coccidiosis, caused by *Eimeria* spp., is a leading factor in enteritis among young camels, leading to high rates of morbidity and mortality during severe outbreaks. Morbidity rates in calf populations can soar to 60–80%, while mortality rates in untreated cases can vary from 5 to 20%, marking it as a significant contributor to pre-weaning losses (38). Those that survive often endure prolonged episodes of diarrhea, which can result in malnutrition, growth retardation, and considerable distress, severely affecting the welfare of juvenile populations (38, 66). The economic significance is associated with treatment costs, lost growth potential, and the mortality of future productive animals (Table 2).

**Table 2:** Major parasitic diseases of dromedary camels: prevalence, clinical signs, and zoonotic potential.

Parasite	Species	Clinical signs	Prevalence (Range)	Zoonotic potential	References
Hemoparasites	*Trypanosoma evansi* (Surra)	Fever, anemia, edema, lethargy, weight loss, abortion	10–30% (seroprevalence can be >50% in endemic areas)	Considered non-zoonotic, but rare human cases have been reported	(2, 57)
	*Anaplasma marginale*	Fever, anemia, jaundice, weight loss	5–20% (varies by region)	Not a primary human pathogen, but related species are	(3, 47)
Ectoparasites	*Sarcoptes scabiei* var. *cameli* (Mange)	Intense pruritus, alopecia, hyperkeratosis, skin thickening	Up to 25% in affected herds; highly contagious	Yes (causes transient scabies in humans)	(111, 112)
	*Hyalomma dromedarii* (Tick)	Skin damage, anemia (heavy infestations), restlessness	Very high (>80% in many pastoral systems)	Vectors for Crimean-Congo Hemorrhagic Fever (CCHF) virus	(57, 72)
Gastrointestinal nematodes	*Haemonchus longistipes*	Anemia, bottle jaw, submandibular edema, weight loss	Prevalence often >60%; high burden common	No	(54, 61)
	*Trichostrongylus* spp.	Diarrhea, weight loss, poor condition	Very common; often part of mixed infections	No	(64, 65)
Protozoan endoparasites	*Eimeria dromedarii* (Coccidiosis)	Diarrhea (sometimes hemorrhagic), dehydration, and mortality in calves	Common in young camels; prevalence 20–50%	No	(3)
Zoonotic helminths	*Echinococcus granulosus*	Often asymptomatic in camels; cysts found in lungs/liver	Variable; 5–15% in slaughterhouse studies	Yes (Causes Cystic Echinococcosis in humans)	(100, 113)

## 4 Helminths of zoonotic importance

Gastrointestinal nematodes represent the primary helminthic concern for the health of camels; however, various cestode (tapeworm) and trematode (fluke) infections pose considerable zoonotic risks, thereby establishing a crucial connection between camel welfare and public health safety. Although the prevalence of these parasites in camels is generally lower than in other ruminants, their existence leads to productivity declines and remains a continuous threat to human populations.

### 4.1 Cestodes (tapeworms)

The most notable zoonotic cestode is *Echinococcus granulosus*, responsible for Cystic Echinococcosis (CE) or hydatid disease. Camels act as intermediate hosts, predominantly containing hydatid cysts within their lungs and liver. Surveys conducted in slaughterhouses within endemic areas, such as the Middle East and North Africa, indicate infection rates in camels that range from 5% to over 20% (67, 68). Although camels often remain asymptomatic, significant cyst loads can result in organ condemnation, leading to direct economic repercussions for the meat industry. The more substantial impact, however, pertains to human health. Humans contract the infection by inadvertently ingesting eggs excreted in the feces of infected definitive hosts, usually dogs that have consumed infected camel offal. CE is classified as a neglected tropical disease that inflicts considerable morbidity and mortality within pastoral communities, with treatment being both complex and expensive (69, 70).

Other cestodes, such as *Moniezia* spp., are prevalent yet non-zoonotic, leading to diarrhea, especially in young camels, which results in diminished growth rates and productivity (71). Although trematode infections in camels are reported less frequently, they include species that possess both direct and indirect zoonotic potential. The liver flukes *Fasciola hepatica* and *F. gigantica* have been identified in camels. The prevalence of these infections varies but can be considerable in regions with appropriate wetland habitats for the intermediate snail host. Such infections lead to liver condemnation, fibrosis, and decreased weight gain. This represents a significant zoonosis, as humans can contract the infection through the ingestion of contaminated aquatic plants, such as watercress (72, 73). *Schistosoma* spp. is capable of infecting camels, resulting in granulomatous lesions in the liver and intestines. Camels may act as reservoir hosts for species like *S. japonicum*, which complicates control measures in endemic regions. Human schistosomiasis is a debilitating condition acquired through exposure to cercariae-infested freshwater (74).

### 4.2 Nematodes (roundworms) with zoonotic potential

In addition to the primary gastrointestinal nematodes, certain species can lead to zoonotic infections. *Trichuris* (whipworm) is generally host-specific; however, close interaction with infected animals presents a 6 impact. The cumulative consequences of polyparasitism lead to a cycle of persistent suffering, economic detriment, and increased susceptibility, thereby jeopardizing the sustainability of livelihoods reliant on camels. An integrated One Health strategy that combines sophisticated diagnostics, established welfare evaluation methods, and customized control measures is critically needed to address these complex issues (75, 76).

## 5 Welfare implications of parasitic diseases

Parasitic infestations create complex welfare challenges for dromedary camels, affecting not only their clinical health but also their behavioral, productive, and socio-economic aspects. These conditions disrupt normal physiological processes, lead to ongoing suffering, and hinder the camel’s ability to flourish within its ecological and cultural environment. Importantly, the effects extend beyond the health of the animals, impacting the livelihoods of pastoralists, the dynamics of human-animal relationships, and public health through zoonotic transmission.

### 5.1 Pathophysiological and affective consequences

Chronic parasitism causes considerable pain, distress, and physical debilitation. Infestations by mange mites (*Sarcoptes scabiei* var. *cameli*) result in severe itching, which can lead to skin damage, lesions, and secondary bacterial infections. Camels suffering from these conditions often display signs of restlessness and social withdrawal, reflecting significant physical and psychological distress (3). Likewise, hemoparasites like *Trypanosoma evansi* cause ongoing anemia, fever, and swelling, which contribute to lethargy and overall weakness (22). The chronic nature of these ailments, frequently exacerbated by delayed interventions in isolated pastoralist communities, results in prolonged periods of aversion and diminished affective well-being. Continuous immune responses to parasitic antigens lead to a catabolic state, worsening weight loss and exhausting energy reserves. This metabolic burden, along with nutrient malabsorption due to gastrointestinal nematodes, results in poor body condition and chronic fatigue, which directly affects the camel’s ability to engage in natural behaviors such as grazing, rumination, and social interaction (77).

### 5.2 Reduced productivity and work capacity

Parasitic diseases significantly reduce essential productive outputs, such as milk yield, meat production, and draft capacity. Trypanosomiasis is especially harmful to work performance due to fatigue caused by anemia (22). Gastrointestinal helminths hinder nutrient absorption, resulting in decreased weight gain and growth stunting, which poses a threat to food security in communities that depend on camels (78). Ectoparasite infestations (for instance, ticks and biting flies) lead to irritability and disrupt feeding and resting patterns, further diminishing productivity and welfare. The reduction in milk production not only impacts income generation but also jeopardizes the nutrition and health of suckling calves, raising concerns about intergenerational welfare.

### 5.3 Reproductive and nutritional deficits

Parasitic infections have a significant detrimental impact on reproductive performance. *T. evansi* is linked to abortion, stillbirth, and decreased conception rates (79). Chronic parasitism leads to poor body condition, which in turn delays puberty and prolongs postpartum anestrus in females, while also diminishing libido and semen quality in males. These issues pose a threat to herd sustainability and the resilience of pastoralists. Furthermore, nutritional status is adversely affected through various mechanisms: blood-feeding parasites (such as *Haemonchus* spp. and ticks) cause anemia and hypoproteinemia; protozoal infections like coccidiosis lead to diarrhea and dehydration, particularly in juveniles (22). The camel’s renowned ability to withstand drought is compromised by hyperthermia, panting, and cutaneous water loss induced by parasites, undermining a crucial adaptive advantage in arid regions (80).

### 5.4 Human-animal interactions and zoonotic risks

**Figure 2:**
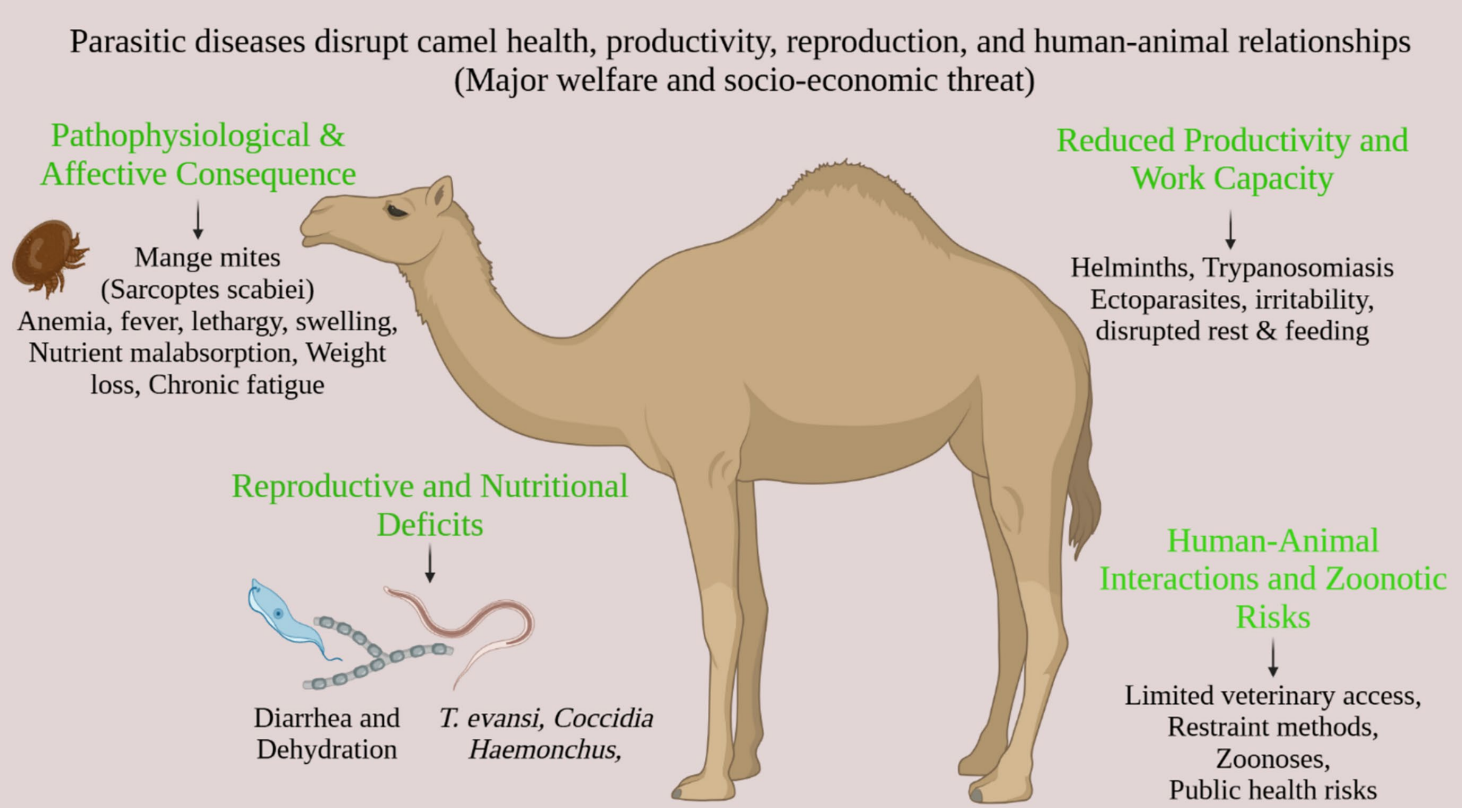
The cascading impacts of parasitic diseases in camels on welfare, productivity, and socio-economic stability. The diagram outlines the pathway from initial infection by specific pathogens (e.g., mange mites, helminths, *Trypanosoma evansi*) through pathophysiological consequences (e.g., anemia, weight loss), to broader outcomes including reduced work capacity, reproductive deficits, and compromised human-animal interactions due to zoonotic risks and limited veterinary care.

The welfare of camels is greatly affected by the responses of their owners to parasitism. Delays in treatment caused by limited access to veterinary care, financial limitations, or a lack of knowledge can prolong the suffering of these animals and may result in the culling of those that are severely weakened (8). Restraint techniques employed during treatment (such as limb-tying and nose-pegging) can cause additional stress and injury if not executed with proper training. Numerous camel parasites pose zoonotic risks, including *Toxoplasma gondii* (found in milk), *Cryptosporidium* spp., *Giardia* spp., and *Echinococcus granulosus* (3). Public apprehension regarding transmission can lead to the stigmatization of camel products, decrease their market value, and result in neglect or improper management practices, which indirectly worsen welfare issues (81). These dynamics underscore the crucial interconnections between animal welfare, public health, and economic stability (Figure 2).

## 6 Gaps in research, surveillance, and veterinary systems

### 6.1 Diagnostic limitations and the welfare consequences of underreporting

The effectiveness of surveillance and timely intervention is severely compromised by limited diagnostic capabilities in remote pastoralist areas (82). Many diagnoses are based on clinical signs or low-sensitivity microscopy, which often fail to identify subclinical cases or co-infections, leading to considerable underreporting and misdiagnosis. This diagnostic gap has direct and severe welfare consequences: delayed or incorrect treatment results in the progression of preventable suffering (20). Animals endure prolonged pain from conditions like mange, chronic debilitation from trypanosomiasis, and ongoing distress from gastrointestinal parasites, all of which could be alleviated with earlier, accurate detection (2). Innovative field-applicable tools such as pen-side serological assays, loop-mediated isothermal amplification (LAMP, a simple, rapid DNA amplification technique that does not require a thermal cycler), and portable sequencing technologies present promising opportunities for enhanced, rapid diagnosis (83, 84). However, their deployment faces significant hurdles, including cost, the need for a stable cold chain, and technical training, which currently limit their practicality in nomadic systems. The development of affordable, robust, and camel-specific point-of-care tests remains a critical unmet need (82, 85).

### 6.2 The impact of climate change on parasite ecology and distribution

Climate change is a significant driver modifying the distribution and intensity of parasitic diseases in camels (3). Rising temperatures and altered precipitation patterns are broadening the geographical range of vectors, such as ticks and biting flies, for pathogens like *Trypanosoma evansi* and Anaplasma spp. (81). Concurrently, desertification and shifts in land use are pushing camel husbandry into new areas, potentially exposing naïve populations to unfamiliar parasite communities (82, 83). Predictive ecological modeling and longitudinal studies are crucial for anticipating these changes and formulating proactive, welfare-focused mitigation strategies, such as targeted parasite control in emerging risk areas (85).

### 6.3 Strengthening veterinary capacity and community engagement

The critical shortage of formal veterinary services in nomadic and pastoralist systems remains a fundamental limitation to improving camel welfare (7). While ethnoveterinary knowledge is valuable and culturally important, it is essential to complement it with evidence-based parasitology and welfare-oriented management practices (82). A key strategy is educating and empowering community-based animal health workers (CAHWs) (86, 87). Training CAHWs in rational drug use, basic diagnostics (e.g., fecal egg counts, recognizing clinical signs of major diseases), and low-stress handling techniques can dramatically improve early detection and enable more humane and timely interventions (88). Such community-level programs bridge the gap between pastoralists and distant veterinary clinics (9), fostering a proactive approach to camel health and welfare (89, 90). Ultimately, increased investment in research and greater policy focus are necessary to elevate camel health (83) and welfare on national and global veterinary and agricultural agendas (80).

## 7 Strengthening diagnostic capacity and surveillance systems

### 7.1 Early diagnosis and surveillance

Accurate and prompt diagnosis serves as the foundation for effective management of parasites. Although microscopy is still extensively utilized in field environments, its shortcomings in identifying subclinical cases or co-infections highlight the need for more sensitive and specific diagnostic tools. Molecular diagnostics, such as PCR, LAMP, and next-generation sequencing, along with serological tests like ELISA, provide enhanced detection capabilities but must be tailored for pathogens specific to camels and the contexts of pastoralists (91). Investing in point-of-care rapid testing, mobile laboratory units, and telemedicine solutions can help close diagnostic gaps in remote regions, facilitating earlier interventions and alleviating chronic suffering (92). Additionally, integrating camel health into national surveillance systems is essential for producing comprehensive epidemiological data and guiding evidence-based control strategies (93).

### 7.2 Vaccination and vector control programs

Currently, there are no commercially available vaccines specifically for camel parasites; however, progress in immunoprophylaxis for similar livestock pathogens offers a promising path. Research into recombinant antigens, viral vector systems, and nanoparticle delivery methods could lead to effective vaccines against major pathogens such as *Trypanosoma evansi* or key tick species (94). Meanwhile, integrated vector management, which includes rotating acaricides, using biological controls like entomopathogenic fungi, and making environmental adjustments, can help reduce ectoparasite populations while addressing concerns about chemical resistance and ecological impacts (21). The targeted use of endectocides, such as ivermectin, should be carefully adjusted to minimize resistance risks and align with seasonal changes in parasite prevalence (95).

### 7.3 Education and training for camel owners and handlers

Improvements in sustainable welfare depend on the knowledge and practices of camel owners and handlers. Educational initiatives involving community participation, delivered through mobile technologies, workshops, and visual aids, can significantly improve understanding of parasite life cycles, zoonotic risks, and early disease detection (83). Training programs for CAHWs should emphasize welfare-sensitive handling, rational drug use, and preventive strategies such as rotational grazing and improved sanitation (96). Empowering local stakeholders promotes early intervention, reduces treatment delays, and encourages culturally sustainable practices.

### 7.4 Welfare-sensitive treatment and handling protocols

Medical and procedural interventions must prioritize the well-being of animals. Stressful restraint techniques (such as forced recumbency and nose-pegging) ought to be substituted with low-stress alternatives that are informed by the ethology of camels (22). Guidelines regarding drug administration, which include correct dosing, withdrawal periods, and the avoidance of toxic combinations, must be widely disseminated to avert iatrogenic harm (97). Likewise, topical applications (for instance, acaricides) should be carefully formulated and applied to reduce cutaneous irritation and systemic side effects.

### 7.5 Policy and legislative support

The welfare of camels is significantly overlooked in livestock policies and global welfare standards. It is crucial to achieve legislative acknowledgment to allocate resources for parasite management, subsidize veterinary services, and facilitate research projects (98, 99). Furthermore, it is vital to incorporate camel health into One Health surveillance systems to effectively tackle zoonotic parasites (such as *Echinococcus granulosus* and *Cryptosporidium* spp.) at the intersection of human, animal, and environmental health (86). As climate change and the expansion of agriculture exacerbate parasitic risks, policy frameworks should foster resilience through strategies that prioritize welfare (Table 3).

**Table 3:** Integrated parasite control strategies in camelid management.

Strategy	Specific approach	Key advantage	Limitation	References
Chemical control	Strategic Anthelmintic Use: Treating based on parasite monitoring (e.g., FEC) rather than a fixed schedule.	Reduces drug resistance and is more cost-effective.	Requires diagnostic capability and owner education.	(114, 115)
	Acaricide Rotation: Rotating chemical classes (e.g., pyrethroids, amitraz) for tick control.	Slows the development of resistance in tick populations.	Logistically challenging in extensive systems; environmental contamination risk.	
Management & environmental	Rotational Grazing: Moving herds to fresh pastures to break parasite life cycles.	Reduces pasture contamination with infective larvae.	Requires sufficient land and management effort; not always feasible in arid zones.	(116, 117)
	Biological Control: Use of entomopathogenic fungi (e.g., Metarhizium anisopliae) against ticks.	Environmentally friendly, target-specific.	Still in experimental stages for field use in camels; cost and application logistics.	
Diagnostic & technological	Point-of-Care Tests: Development of rapid tests for *T. evansi* or Fecal Egg Count (FEC) kits.	Enables early diagnosis and timely treatment in remote areas.	Limited commercial availability of camel-specific tests.	(118, 119)
	Vaccine Development (Future)	Research into vaccines against *T. evansi* or tick antigens.	Would provide sustainable, long-term protection.	
Community-based	Community Animal Health Workers (CAHWs): Training local handlers in basic health care and welfare assessment.	Improves access to basic care and early detection in remote pastoral systems.	Sustainability depends on continuous support and training.	(96)

## 8 Conclusion

This review has synthesized evidence from veterinary parasitology and animal welfare science to elucidate the profound, multifaceted impact of parasitic diseases on dromedary camels. Our analysis demonstrates that pathogens such as *Trypanosoma evansi* (with seroprevalence exceeding 50% in endemic areas), mange mites (affecting up to 25% of herds), and gastrointestinal nematodes (prevalence >70%) are not merely production constraints but direct causes of significant suffering (22). These infections systematically compromise all domains of welfare, leading to chronic pain, debilitating anemia, severe pruritus, and behavioral distress, which in turn result in substantial production losses (e.g., up to 40% reduction in milk yield, 15–30% decrease in meat production) (23). The situation is exacerbated by critical gaps in species-specific welfare assessment, diagnostic capabilities in pastoralist systems, and a fundamental underappreciation of the affective states of camels (3, 100). Furthermore, the presence of zoonotic parasites like *Echinococcus granulosus* creates an inseparable link between camel welfare and public health, necessitating an integrated One Health/One Welfare perspective (101).

## 9 Recommendations and future directions

To address these challenges, a coordinated, multi-stakeholder approach is urgently required. There is a critical need to develop and validate a set of practical, animal-based welfare indicators specific to dromedary camels for use in field conditions (88). Capacity must be strengthened by training CAHWs in low-stress handling, rational anthelmintic use, and the recognition of common parasitic diseases (94). Awareness campaigns should be implemented to educate pastoralists on the life cycles of parasites, zoonotic risks, and the economic and welfare benefits of early intervention (102). Future research should focus on developing affordable, point-of-care diagnostic tests for key pathogens, such as *T. evansi* (11). Investigations into the pathophysiology of parasitism should be expanded to better quantify the pain and distress associated with infections such as sarcoptic mange and trypanosomiasis (102). Long-term studies are necessary to understand the impacts of climate change on parasite distribution and to explore the potential for vaccine development against major parasites (20). Policy frameworks should formally incorporate camel welfare and parasitic disease control into national animal health strategies and One Health surveillance programs. Economic incentives, such as sustainable branding of parasite-free or high-welfare camel products, should be explored to reward good management practices. International collaboration and funding must be prioritized to support research and implementation programs in regions dependent on camel husbandry.

In summary, this review establishes that parasitic diseases are a central, yet often neglected, animal welfare crisis in dromedary camels. By moving beyond a purely production-centric view and integrating the principles of One Welfare, we can reframe effective parasite control as a moral imperative. The path forward requires translating this integrated understanding into practical tools, empowered communities, and supportive policies to safeguard the well-being of these essential animals, the livelihoods of the people who depend on them, and the public health of us all.
